# Seizure evoked regulation of LIM-HD genes and co-factors in the postnatal and adult hippocampus

**DOI:** 10.12688/f1000research.2-205.v1

**Published:** 2013-10-04

**Authors:** Vanisha Lakhina, Lakshmi Subramanian, Dhananjay Huilgol, Ashwin S Shetty, Vidita A. Vaidya, Shubha Tole

**Affiliations:** 1 Department of Biological Sciences, Tata Institute of Fundamental Research, Mumbai, India; 2 Current affiliation: Lewis Sigler Institute for Integrative Genomics, Princeton University, NJ, USA; 3 Current affiliation: Department of Neurology, University of California, San Francisco, CA, USA; 4 Current affiliation: Cold Spring Harbor Laboratory, NY, USA

## Abstract

The LIM-homeodomain (LIM-HD) family of transcription factors is well known for its functions during several developmental processes including cell fate specification, cell migration and axon guidance, and its members play fundamental roles in hippocampal development. The hippocampus is a structure that displays striking activity dependent plasticity.  We examined whether LIM-HD genes and their co-factors are regulated during kainic acid induced seizure in the adult rat hippocampus as well as in early postnatal rats, when the hippocampal circuitry is not fully developed.  We report a distinct and field-specific regulation of LIM-HD genes *Lhx1,* *Lhx2*, and *Lhx9*, LIM-only gene *Lmo4*, and cofactor *Clim1a *in the adult hippocampus after seizure induction. In contrast none of these genes displayed altered levels upon induction of seizure in postnatal animals.  Our results provide evidence of temporal and spatial seizure mediated regulation of LIM-HD family members and suggest that LIM-HD gene function may be involved in activity dependent plasticity in the adult hippocampus

## Introduction

Transcription factors regulate gene expression in the mammalian brain, playing a critical role in both neurodevelopment and in neuronal plasticity during its lifespan. During development, transcription factor mediated regulation is essential for appropriate cell fate specification, cell migration and connectivity ^1– 3^. Transcription factors also regulate plasticity including activity-dependent process of dendritic pruning, axonal sprouting and cell proliferation and survival ^4– 6^.

One family of transcription factors, the LIM-homeodomain (LIM-HD) family, is known to play critical roles in regulating cell proliferation, axon outgrowth and pathfinding across several systems ^7– 11^. The LIM-HD proteins have a C-terminal homeodomain which binds to DNA and two zinc finger “LIM” domains that bind co-factors encoded by the *Clim* genes. The transcriptionally active complex is a tetramer comprising two LIM-HD molecules bridged by a dimer of two Clim molecules ^12, 13^. LIM-only (Lmo) proteins lack the homeodomain but can bind Clim molecules, and function as dominant-negative regulators of LIM-HD function ^12, 14, 15^. At least thirteen LIM-HD ( *Lhx*) genes four *Lmo* genes and two *Clim* genes have been identified in the mouse. A subset of genes is expressed in the embryonic and mature hippocampus and of these, *Lhx2* and *Lhx5* are critical to hippocampal development ^16^. *Lhx2* plays a fundamental role in early telencephalic development as a cortical selector gene ^11^. The neocortex and hippocampus do not form in the absence of *Lhx2* ^11, 17^. At later stages, *Lhx2* plays a new role in the developing hippocampus, as a necessary and sufficient repressor of astrogliogenesis ^18^. *Lhx2* continues to be expressed in the mature hippocampus. *Lhx5* is critical for hippocampal development at early stages, but is not expressed in the embryonic hippocampus once it is specified ^19^. *Lhx1*, *Lhx9*, *Clim1a*, *Clim2*, *Lmo3* and *Lmo4* are all expressed in the hippocampus at embryonic and adult stages ^16^, but no loss of function phenotypes have been reported in the hippocampus.

While several studies have implicated the LIM-HD family as a key modulator of important neurodevelopmental events, the understanding of the role of this transcription factor family in the postnatal and adult brain remains relatively unexplored. These transcription factors are known to regulate cell proliferation ^8, 20^, axon pathfinding ^21, 22^ and neurite outgrowth ^23, 24^. These phenomena have parallels in the structural plasticity that occurs in postnatal and adult life. It is now well established that the same molecules that bring about the early development of the hippocampus are often reutilized in adult reorganization and structural plasticity ^25– 27^. Several LIM-HD family members continue to be expressed in the adult hippocampus ^16^ (this study). Therefore, we explored whether these genes display activity-dependent regulation in the adult hippocampus, to provide a basis for studies that may uncover new functions for these genes in maturity.

Activity dependent neuronal plasticity has been suggested to reutilize key developmental pathways to evoke plasticity in the mature nervous system. In particular, seizure models have been shown to induce dramatic changes in progenitor proliferation, axonal sprouting, dendritic reorganization, changes in neuronal cell survival and progenitor differentiation within the hippocampus ^28– 31^. Intriguingly, the nature of neuroplastic changes evoked by seizures differs quite dramatically in the postnatal versus the adult brain ^32– 34^. Regulation at the level of signaling and transcription factors has been shown to be important for structural plasticity in the hippocampus ^35^. While neuronal activity and seizures are likely to recruit major developmental signalling pathways in the hippocampus, thus far the role of key developmental transcription factor families as targets is relatively unexplored.

An earlier study reported that LIM-only genes *Lmo1, 2* and *3* are differentially regulated in a field-specific manner in the adult rat hippocampus in response to kainic acid-induced seizure ^36^. We examined a broader set of Lmo and LIM-HD genes as well as their co-factors in a similar paradigm, not only in the adult rat hippocampus, but also in early postnatal stages when hippocampal circuitry is not fully developed ^37– 39^. Our study provides evidence that LIM-HD, LIM-only, and Clim gene mRNA displays selective field-specific regulation in the hippocampus in response to kainate induced seizures. This provides a basis to explore potential new functions of these genes in activity-dependent synaptic plasticity.

## Results

In this study we focused on LIM-HD genes that are expressed in the adult hippocampus, *Lhx1, Lhx2* and *Lhx9* and their co-factors, *Clim1a* and *Clim2*. Among the LIM-only genes, *Lmo1, Lmo2*, and *Lmo3* have been previously reported to display differential regulation in kainate-induced seizure ^36^. In our study, we included *Lmo3* as a control to allow comparison with the earlier study ^36^, and also *Lmo4* which was not examined previously. We examined the mRNA expression of these genes at postnatal day P7 when the hippocampal circuitry is not yet fully developed, and also in adult rats (2–3 months old) with mature hippocampal neurocircuitry.

### Differential expression of LIM family members and their co-factors across different hippocampal fields

**Figure 1.**
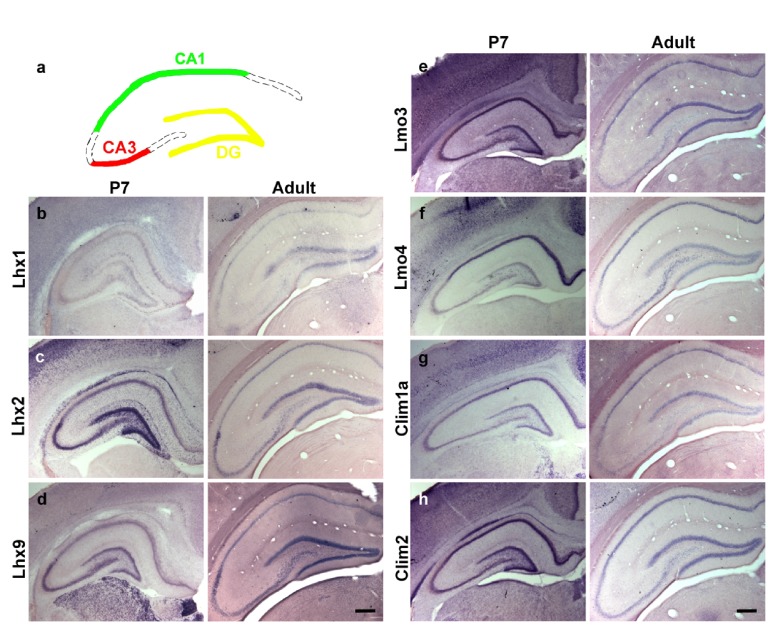
Expression of LIM genes and co-factors in early postnatal and adult hippocampus. ( **a**) A schematic illustrating hippocampal subfields; dentate gyrus (DG) (yellow), CA3 (red) and CA1 (green) fields of Ammon’s horns. ( **b–d**) Non-radioactive *in-situ* hybridization of LIM-homeodomain genes at postnatal day (P)7 and in adult control animals showing differential expression of LIM-homeodomain genes; *Lhx1* ( **b**), *Lhx2* ( **c**) and *Lhx9* ( **d**), *Lmo3* ( **e**), *Lmo4* ( **f**) *Clim1a* ( **g**), *Clim2* ( **h**) across the hippocampal subfields. Scale bars = 200µm

We used non-radioactive *in-situ* hybridization to examine gene expression in the CA1 and CA3 fields of the Ammon’s horn as well as the dentate gyrus (DG) of control animals ( Figure 1a). *Lhx1* transcripts were not detectable in the hippocampus at P7, and only weakly expressed in the adult DG ( Figure 1b). In contrast, *Lhx2* and *Lhx9* are expressed intensely in the DG and CA3, with weaker expression in CA1 at P7. In the adult, expression was strong in the DG, but weak in CA1 and CA3 ( Figure 1c, d). *Lmo3* and *Clim2* are strongly expressed in CA1 and DG, with weaker expression in the CA3 region at both stages ( Figure 1e, h). *Lmo4* shows strong expression in CA1 but is weakly expressed in CA3 and DG at both stages ( Figure 1f). *Clim1a* displays expression in all fields at P7, but is weak to undetectable in CA3 in the adult ( Figure 1g).

Activity is known to regulate structural plasticity and neurogenesis in the adult hippocampus ^40, 41^. We administered kainate intraperitoneally to both early postnatal and adult rats to induce seizures as a model of activity and analysed whether there is differential regulation of LIM genes in response to kainate-evoked seizures 6 hours later. All animals administered kainate exhibited classical hallmarks of seizure. Using radioactive *in-situ* hybridization and optical densitometry we assessed the expression of *Lhx1, Lhx2*, *Lhx9*, *Lmo3, Lmo4, Clim1a* and *Clim2* in the postnatal and adult hippocampal subfields (see Materials and methods). Radioactive *in-situ* hybridization has an important advantage over quantitative PCR since it provides spatial resolution. The hippocampal CA1 and CA3 fields are molecularly distinct, and the dentate gyrus contains a distinct cell population from the Ammon’s horn ^42^. Therefore it is necessary to quantitate the gene expression in each region individually.

### Seizure induced regulation of LIM family members and their co-factors in the adult dentate gyrus (DG)

**Figure 2.**
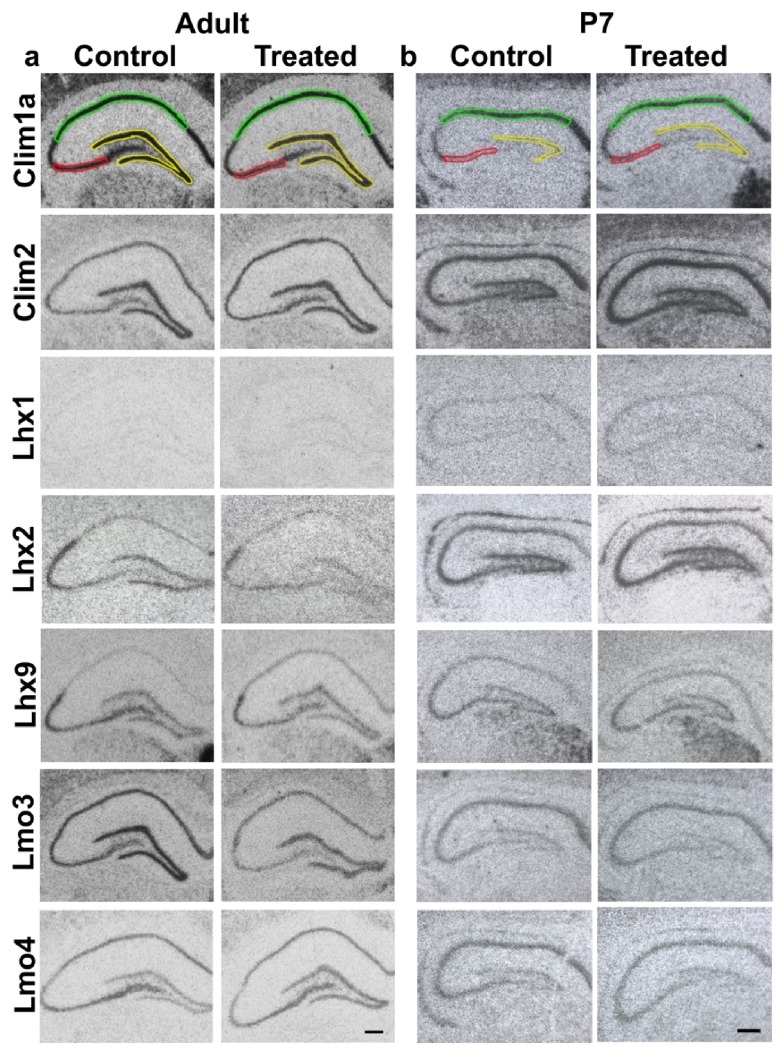
Expression of LIM genes and co-factors used for densitometric analysis. Representative images of sections of brains from control and kainate-administered animals processed for radioactive *in-situ* hybridization of LIM-homeodomain genes in the hippocampus. 1 section from each condition is shown for adult ( **a**) and P7 ( **b**) animals. Colored lines mark the areas for quantification of expression in different hippocampal subfields: DG (yellow); CA3 (red); CA1 (green). Scale bars = 200µm.

**Figure 3.**
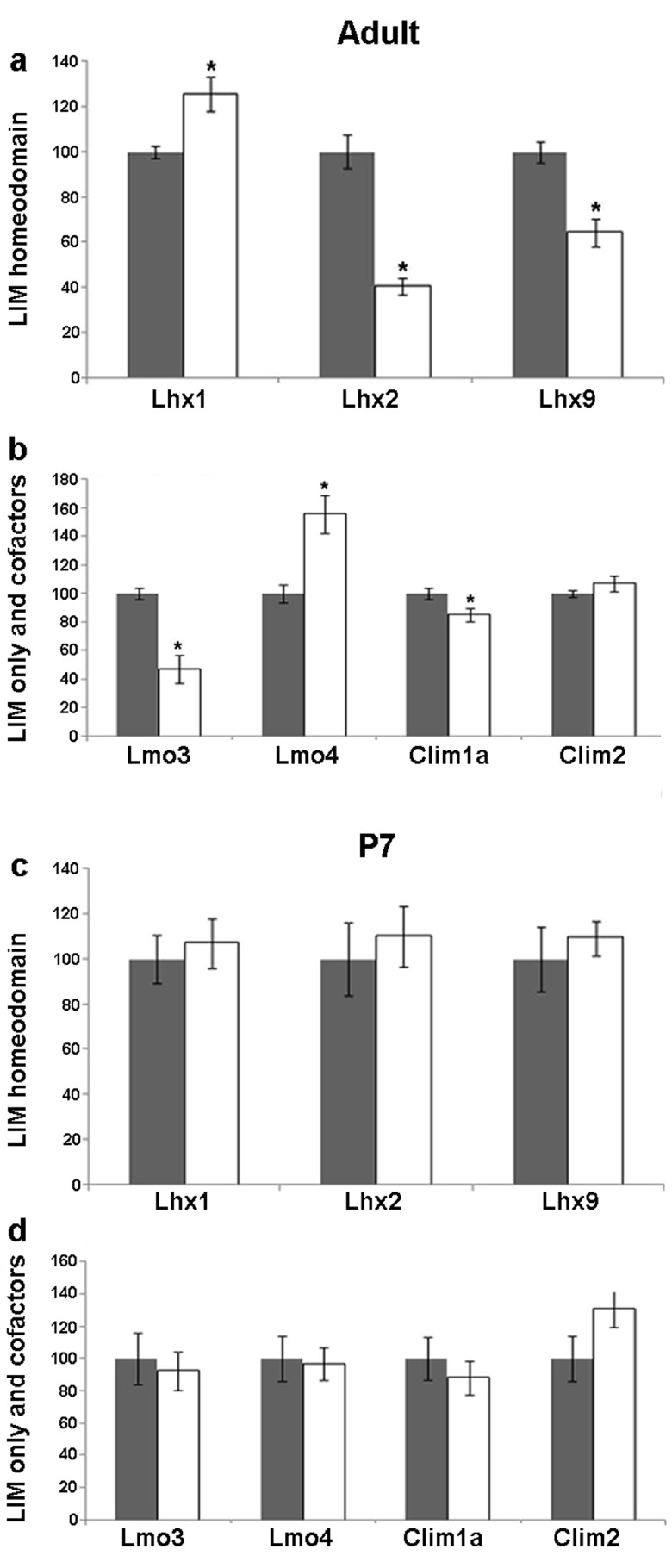
Kainate-induced regulation of LIM genes and co-factors in the dentate gyrus (DG) of adult and P7 rats. Quantitative densitometric analysis of the adult DG region following kainate administration in adult ( **a**, **b**) and P7 ( **c**, **d**) rats. Grey bars are controls, white bars are kainate treated animals. Results are expressed as mean ± SEM percentage of control for mRNA expression (* *p* < 0.05, unpaired Student’s *t* test).

The DG displays robust structural changes in response to seizure. Increase in dentate granule cell neurogenesis ^43^ and extensive mossy fiber sprouting ^41, 44^ are hallmarks of kainate induced seizure. Upon kainate treatment, the expression of *Lhx1* showed a striking increase (25%; p = 0.019) in the adult DG. This is in contrast with the adult *Lhx2* and *Lhx9* expression, the mRNA levels of which show a drastic reduction (60% for *Lhx2*, p = 0.0004 and 36% for *Lhx9*, p = 0.003; Figure 2, Figure 3a). Interestingly, the LIM-only genes *Lmo3* and *Lmo4* also showed opposite changes: whereas *Lmo3* levels decreased significantly (53%, p = 0.002), *Lmo4* mRNA levels showed a remarkable increase (55%, p = 0.009) in kainate-treated animals. The decrease in *Lmo3* levels was consistent with that reported previously ^36^. The mRNA levels of the cofactor *Clim1a* decreased slightly in treated animals (15%, p = 0.048) whereas no significant difference was observed with *Clim2* ( Figure 2, Figure 3b).

### Seizure induced regulation of LIM family members and their co-factors in the adult CA3 subfield

**Figure 4.**
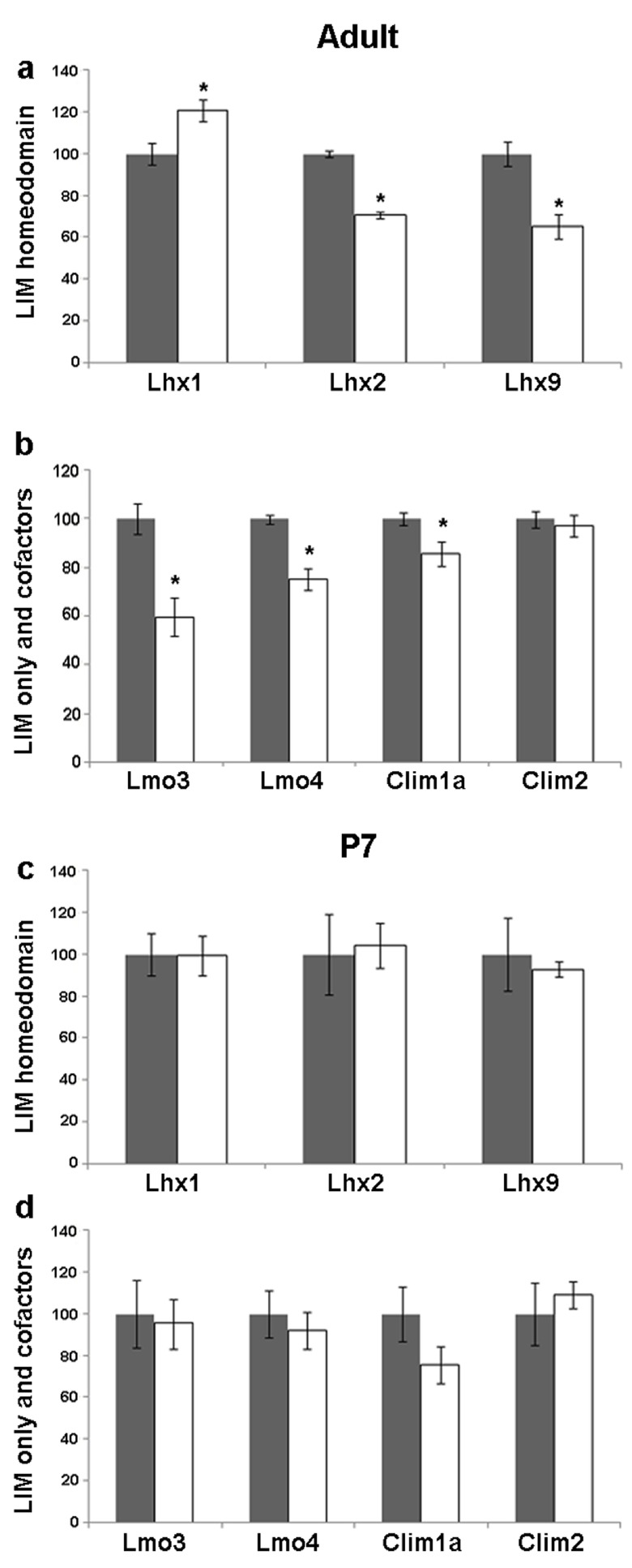
Kainate-induced regulation of LIM genes and co-factors in CA3 of adult and P7 rats. Quantitative densitometric analysis of the adult CA3 region following kainate administration in adult ( **a**, **b**) and P7 ( **c**, **d**) rats. Grey bars are controls, white bars are kainate treated animals. Results are expressed as mean ± SEM percentage of control for mRNA expression (* *p* < 0.05, unpaired Student’s *t* test).

The CA3 subfield has pyramidal neurons, which receive input from the dentate granule cells. They display profound alterations in dendritic structure and branching in response to seizure. In our experiments using kainate-induced seizure, *Lhx1* mRNA increased (20%, p = 0.014) in the adult CA3. In contrast, *Lhx2* and *Lhx9* levels decreased (30%, p = 0.028; 35%, p = 0.044 respectively; Figure 2, Figure 4a). Levels of both *Lmo3* and *Lmo4* were reduced (40%, p = 0.007; 25%, p = 0.002 respectively). The levels of the cofactor *Clim1a* also decreased (15%, p = 0.047) whereas *Clim2* levels remained unaltered in the adult CA3 ( Figure 2, Figure 4b).

### Seizure induced regulation of LIM family members and their co-factors in the adult CA1 subfield

**Figure 5.**
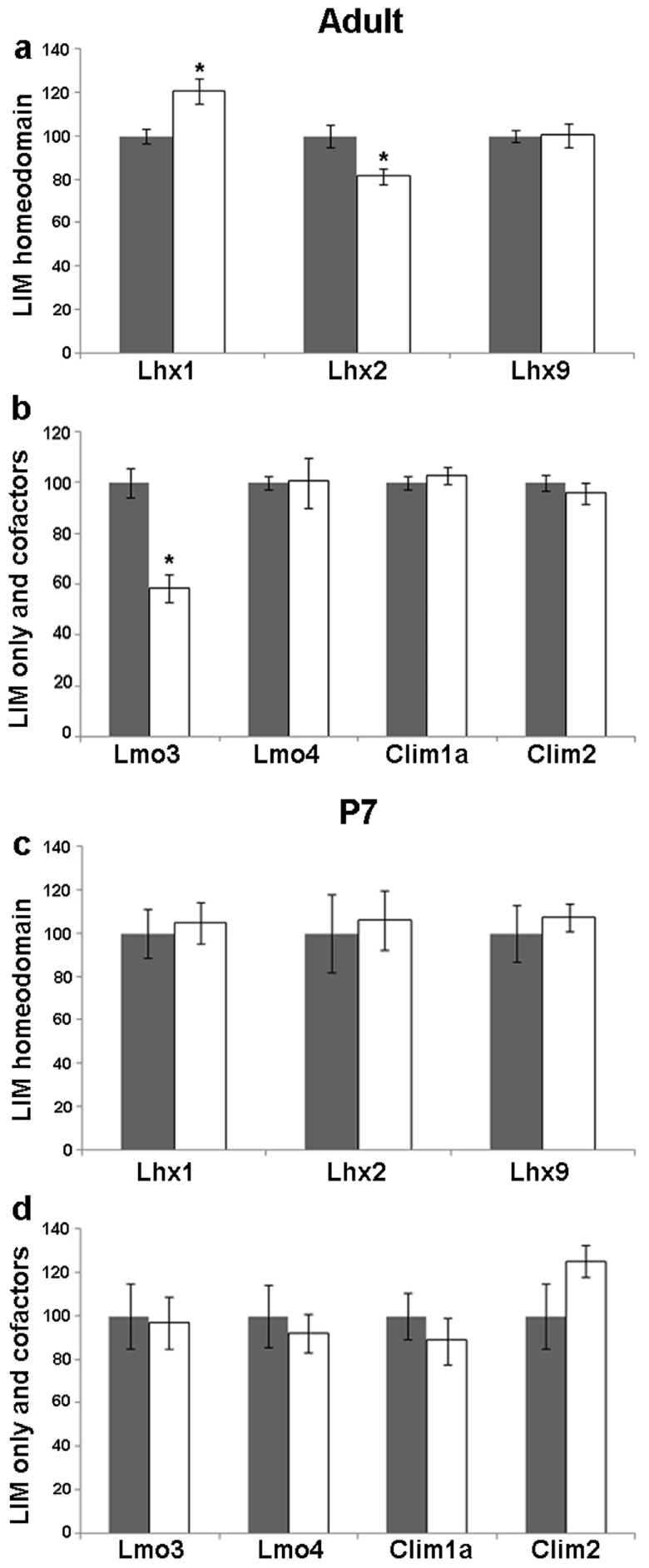
Kainate-induced regulation of LIM genes and co-factors in CA1 of adult and P7 rats. Quantitative densitometric analysis of the adult CA1 region following kainate administration in adult ( **a**, **b**) and P7 ( **c**, **d**) rats. Grey bars are controls, white bars are kainate treated animals. Results are expressed as mean ± SEM percentage of control for mRNA expression (* *p* < 0.05, unpaired Student’s *t* test).

The CA1 pyramidal neurons receive input from the CA3 neurons. They displayed altered dendritic shape and density and also axon sprouting as a result of seizure ^45^. In the CA1 field, *Lhx1* mRNA increased (20%, p = 0.02), whereas *Lhx2* levels decreased (19%, p = 0.024) but, there was no change in *Lhx9* mRNA levels in the adult CA1 upon kainate-induced seizure ( Figure 2, Figure 5a). *Lmo3* mRNA levels decreased (41%, p = 0.0018) whereas *Lmo4*, *Clim1a*, and *Clim2* levels remained unchanged ( Figure 2, Figure 5b).

### A summary of seizure induced regulation of LIM family members and their co-factors across all hippocampal fields

Table 1 summarizes the data such that seizure-induced regulation can be compared within a particular field as well as for a particular gene across all fields. For example, upon kainate induced seizure, *Lhx1* mRNA shows a significant increase over very low baseline expression in all the hippocampal fields in response to seizure. In contrast, *Lhx2* and *Lmo3* show a significant decrease in all hippocampal fields. Interestingly, *Lhx9* and *Clim1a* show a significant decrease in CA3 and DG, but not in CA1. *Lmo4* transcript levels increase in the DG, decrease in the CA3 and show no change in the CA1. This correlates with the fact that the DG and CA3 undergo a more drastic structural reorganization in response to seizure ^46, 47^. *Clim2* shows no alteration suggesting it may not have any additional roles in kainate-induced plasticity, but continues to be available to LIM-HD transcription factors at the same levels.

**Table 1.** Summary of seizure evoked regulation of LIM genes and co-factors across the hippocampal fields.

LIM gene	DG		CA3		CA1	
	Adult	P7	Adult	P7	Adult	P7
**Lhx1**	↑	_	↑	_	↑	_
**Lhx2**	↓	_	↓	_	↓	_
**Lhx9**	↓	_	↓	_	_	_
**Lmo3**	↓	_	↓	_	↓	_
**Lmo4**	↑	_	↓	_	_	_
**Clim1a**	↓	_	↓	_	_	_
**Clim2**	_	_	_	_	_	_

### Seizures do not affect LIM-gene expression in the postnatal hippocampus

Seizure evoked structural plasticity differs between the postnatal and adult hippocampus in its extent as well as the type of changes seen. Although postnatal kainate treatment evokes powerful seizures, the immature brain is relatively resistant to seizure-evoked structural remodeling. For example, mossy fiber sprouting is absent or delayed ^48– 50^, and DG neurogenesis is unaltered or biphasically regulated with an initial decline and a delayed increase ^51– 53^ in response to seizure in the postnatal hippocampus. We asked whether the postnatal hippocampus differs from the adult hippocampus in kainic acid induced regulation of LIM genes and co-factors. We administered kainic acid to rat pups on postnatal day P7 and analyzed changes in transcript levels of several LIM genes 6 hours later. In striking contrast to the changes observed in the adult brain, the postnatal hippocampus appears refractory to regulation of the LIM-HD family following kainate evoked seizures ( Figure 2, Figure 3c, d, Figure 4c, d, Figure 5c, d).

In summary, the LIM gene family and its co-factors display distinct and highly field-specific regulation in response to kainate induced seizure in the adult, but not in the postnatal hippocampus.

Seizure evoked regulation of LIM genes in the hippocampus Data figure. Expression of LIM genes and co-factors used for densitometric analysis Representative images of sections of brains from control and kainate-administered animals processed for radioactive in-situ hybridization of LIM-homeodomain genes in the hippocampus (in addition to Figure 2). Two sections from each condition are shown for adult and P7 animals.
Dataset 1. Quantitations for Clim1a in the adult hippocampus Quantitations from densitometric analysis of hippocampal areas marked in Figure 2a from sections of brains from control and kainate-administered (treated) animals processed for radioactive in-situ hybridization of Clim1a in the adult hippocampus.
Dataset 2. Quantitations for Clim2 in the adult hippocampus Quantitations from densitometric analysis of hippocampal areas marked in Figure 2a from sections of brains from control and kainate-administered (treated) animals processed for radioactive in-situ hybridization of Clim2 in the adult hippocampus.
Dataset 3. Quantitations for Lhx1 in the adult hippocampus Quantitations from densitometric analysis of hippocampal areas marked in Figure 2a from sections of brains from control and kainate-administered (treated) animals processed for radioactive in-situ hybridization of Lhx1 in the adult hippocampus.
Dataset 4. Quantitations for Lhx2 in the adult hippocampus Quantitations from densitometric analysis of hippocampal areas marked in Figure 2a from sections of brains from control and kainate-administered (treated) animals processed for radioactive in-situ hybridization of Lhx2 in the adult hippocampus.
Dataset 5. Quantitations for Lhx9 in the adult hippocampus Quantitations from densitometric analysis of hippocampal areas marked in Figure 2a from sections of brains from control and kainate-administered (treated) animals processed for radioactive in-situ hybridization of Lhx9 in the adult hippocampus.
Dataset 6. Quantitations for Lmo3 in the adult hippocampus Quantitations from densitometric analysis of hippocampal areas marked in Figure 2a from sections of brains from control and kainate-administered (treated) animals processed for radioactive in-situ hybridization of Lmo3 in the adult hippocampus.
Dataset 7. Quantitations for Lmo4 in the adult hippocampus Quantitations from densitometric analysis of hippocampal areas marked in Figure 2a from sections of brains from control and kainate-administered (treated) animals processed for radioactive in-situ hybridization of Lmo4 in the adult hippocampus.
Dataset 8. Quantitations for Clim1a in P7 hippocampus Quantitations from densitometric analysis of hippocampal areas marked in Figure 2b from sections of brains from control and kainate-administered (treated) animals processed for radioactive in-situ hybridization of Clim1a in P7 hippocampus. Dataset 9. Quantitations for Clim2 in P7 hippocampus Quantitations from densitometric analysis of hippocampal areas marked in Figure 2b from sections of brains from control and kainate-administered (treated) animals processed for radioactive in-situ hybridization of Clim2 in P7 hippocampus.
Dataset 10. Quantitations for Lhx1 in P7 hippocampus Quantitations from densitometric analysis of hippocampal areas marked in Figure 2b from sections of brains from control and kainate-administered (treated) animals processed for radioactive in-situ hybridization of Lhx1 in P7 hippocampus.
Dataset 11. Quantitations for Lhx2 in P7 hippocampus Quantitations from densitometric analysis of hippocampal areas marked in Figure 2b from sections of brains from control and kainate-administered (treated) animals processed for radioactive in-situ hybridization of Lhx2 in P7 hippocampus.
Dataset 12. Quantitations for Lhx9 in P7 hippocampus Quantitations from densitometric analysis of hippocampal areas marked in Figure 2b from sections of brains from control and kainate-administered (treated) animals processed for radioactive in-situ hybridization of Lhx9 in P7 hippocampus.
Dataset 13. Quantitations for Lmo3 in P7 hippocampus Quantitations from densitometric analysis of hippocampal areas marked in Figure 2b from sections of brains from control and kainate-administered (treated) animals processed for radioactive in-situ hybridization of Lmo3 in P7 hippocampus.
Dataset 14. Quantitations for Lmo4 in P7 hippocampus Quantitations from densitometric analysis of hippocampal areas marked in Figure 2b from sections of brains from control and kainate-administered (treated) animals processed for radioactive in-situ hybridization of Lmo4 in P7 hippocampus.

## Discussion

### Differential regulation of LIM-gene expression in response to seizures

Seizures can lead to different forms of hippocampal plasticity, which include axonal/dendritic remodeling and neurogenesis. Chemical-induced seizures like the kainic acid (kainate) treatment are used as models for epilepsy and have been shown to increase neurogenesis in the adult DG ^28^ and extensive mossy fiber sprouting where mossy fibers aberrantly synapse onto dentate granule cells instead of CA3 pyramidal neurons ^41, 44^. Kainic acid administration causes animals to display motor signs including convulsions. In our experiments, we observed changes in the transcript levels 6 hours post kainic acid administration, after the animals displayed all the characteristic physical stages of seizures. In future experiments it would be interesting to examine whether any LIM gene transcript regulation occurs in a shorter time window post kainic acid administration, prior to the physical manifestation of seizure by the animal.

Transcription factors important for brain development are also known to regulate structural changes and reorganization in the adult brain, one example being members of the basic Helix-Loop-Helix (bHLH) family ^25, 26^. Members of the LIM-HD family of transcription factors are necessary for different aspects of the development of the hippocampus ^11, 18, 19^, a structure that is vulnerable to changes in response to activity. LIM genes are differentially expressed in both the postnatal and adult hippocampus, suggesting that there might be a role for these genes in postnatal circuit development and adult reorganization ^16^. We therefore hypothesized that the LIM-HD family members are differentially regulated in response to activity. Indeed, from our analysis of radioactive *in-situ* hybridization, we find that each hippocampal field displays differential expression and post-seizure regulation of different LIM genes. ** LIM-only genes *Lmo1*, *2* and *3* were previously shown to be regulated in response to kainate-induced seizures in the adult hippocampus ^36^. We report that *Lmo4* is also regulated by kainate-induced seizures throughout the hippocampus. We also discovered that LIM-HD genes *Lhx1, Lhx2*, *Lhx9* and cofactor *Clim1a* are differentially regulated in response to seizures in a field-specific manner. Furthermore, we show that this differential regulation of LIM genes is restricted to adult animals and when we administered kainic acid to postnatal pups, no such regulation was observed. This is intriguing because these results highlight that a developing system such as the hippocampal circuitry in the early postnatal brain is relatively resistant to seizure-induced structural remodelling and plasticity ^32^. For example, in the adult, seizure induces an increase in DG neurogenesis whereas in early postnatal stages, it is either decreased or unchanged ^34^. Our results raise the intriguing possibility that such differences in molecular regulation of transcription factors may underlie the differing nature of cellular changes evoked by seizures in the postnatal versus adult brain.

### Structural changes in the hippocampus

Seizure leads to an increase in neuronal activity thereby inducing the transcription of several immediate early genes (IEGs). The IEGs are hypothesized to be involved in seizure-induced structural remodelling ^54^. The LIM family of transcription factors could be part of effector cascades downstream of these IEGs, which may eventually lead to the structural changes seen in different hippocampal subfields. CREB, a well-known activity regulated transcription factor, has been shown to interact in the same transcriptional complex as Lmo4 in response to activity ^55^. It is also interesting to note that well known seizure-responsive IEGs in the adult hippocampus, such as the AP-1 complex, are not regulated by postnatal seizures ^56^. This further supports the idea that distinct molecular changes evoked by postnatal versus adult seizures may contribute to the age-dependent differences in seizure-evoked plasticity.

Distinct structural changes occur in response to seizure in different subfields of the hippocampus. On seizure induction, DG shows an increase in the granule cell neurogenesis ^46^, enhanced integration of granule cells into the neurocircuitry, a profound increase in mossy fiber sprouting by these neurons and formation of recurrent synapses ^57– 59^. The CA3 and CA1 pyramidal neurons show a loss of dendritic spine and dendritic branches ^47^ post seizure. Some axon sprouting is also seen in CA1 neurons ^45, 60^. LIM genes may bring about activity induced structural changes in the hippocampus. They are known to regulate neurite outgrowth ^24^. Some LIM-HD genes also control key axon guidance molecules such as Eph/ephrins ^61^, which affect mossy fiber sprouting in the DG ^62^. Lhx1 is known to regulate the transcription of Eph/ephrins in a subset of motor neurons ^61^. Our results show increased *Lhx1* mRNA levels in the DG in response to seizure that could lead to increased Eph/ephrin levels therefore contributing to mossy fiber sprouting. Lhx2 represses Robo1 and 2 expression in the thalamus during thalamocortical pathfinding ^22^ and so down regulation of *Lhx2* mRNA in response to seizure could be important for mossy fiber sprouting via upregulation of the Robo receptors. Lmo4 has been shown to confer a neuroprotective role in response to hypoxia ^63^. Interestingly, we find an increase in the *Lmo4* mRNA after kainate treatment, which could lead to neuronal survival in response to seizure.

Our study provides new evidence of seizure mediated regulation of LIM-HD transcription factors. We show that this regulation is age-dependent and field specific. Future experiments will aim at testing whether LIM genes are necessary for mediating seizure induced structural alterations. Examining the effect of kainic acid treatment on structural changes such as DG neurogenesis in LIM gene loss-of-function mutants will begin to address this issue. In addition, determining the interactions of LIM gene family proteins with other factors known to mediate structural changes such as the bHLH family members ^25, 26^ will open avenues for the mechanistic understanding of this process. These results therefore provide impetus for future studies to explore the role of the LIM-HD transcription factors, LIM only genes, and their cofactors in activity-dependent reorganization and plasticity in the mature nervous system.

## Materials and methods

### Animals and treatment paradigm

Sprague-Dawley rats were bred in the Tata Institute of Fundamental Research (TIFR) Animal house, maintained under normal 12-hour light/dark cycle and were provided with food and water *ad libitum*. A total of 84 adults and 101 pups (P7) were used. Adults were between 2–3 months old and weighed between 200–250 grams. All animal procedures were performed in accordance with the NIH guidelines for use and maintenance of animals and were approved by the TIFR Institutional Animal Ethics committee. The male rats were sexed at P21 and were used for experiments when they reached adulthood. Postnatal pups of both sexes were used for experiments at P7. All animals were grouped based on their treatment with either saline (control group; n = 44 adults; n = 47 P7 pups) or with 10mg/kg kainic acid (Sigma, USA; n = 40 adults; n = 54 P7 pups) administered intraperitoneally and were housed isolated for 6 hours after the treatment. The kainic acid treated group was observed every 30 minutes across the 6 hours and displayed all the characteristic stages of seizures. The animals displayed facial clonus (Racine Stage 1) to front and hindlimb clonus and continuous falling down (Racine Stage 5).

Animals were decapitated using a guillotine 6 hours after treatment and the brains were immediately frozen on dry ice and stored at -70°C. Coronal sections (14µm) were generated on the cryostat and mounted onto Probe-plus RNase free slides (Electron Microscopy Sciences, USA). Slides were then treated with 4% paraformaldehyde (PFA; Merck Chemicals), washed in 1X phosphate-buffered saline, acetylated with acetic acid (Qualigens Fine Chemicals) in 0.1M triethanolamine (Sigma-Aldrich), rinsed in 2X sodium saline citrate (SSC), pH 4.5 and then dehydrated through grades (30%, 70% and 100% in double distilled water) of ethanol (Commercial Alcohols, Ontario, Canada) prior to storage at -70°C.

### mRNA *in-situ* hybridization

The **in-situ** hybridization for DIG-labeled probes was carried out as described previously (Bulchand *et al.*, 2003) ^16^. Plasmid DNAs encoding different LIM genes and co-factors were linearized by restriction digestion to provide template for making DIG-labeled RNA probe ^16^. Briefly, the slides were incubated in hybridization buffer (50% formamide, 5X SSC and 1% SDS) containing DIG-labeled riboprobes (Roche) for 16 hours at 70°C followed by post-hybridization washes using Solution X (50% formamide, 2X SSC and 1% SDS), 2X SSC and 0.2X SSC.

Radioactive *in-situ* hybridization was carried out as described previously ^64^. Briefly, the slides were incubated in the hybridization buffer (50% formamide, 0.6M sodium chloride, 10mM Tris pH 7.4, 1X Denhardts solution, 10mM dithiotheritol (DTT), 250µg/ml yeast tRNA, 50µg/ml Salmon sperm DNA, 10% Dextran sulphate) containing ^35^ S-UTP labeled riboprobes (Amersham, Buckinghamshire, UK) at a concentration of 10 ^6^cpm/250µl for 20–24 hours at 60°C. Post-hybridization, the slides were washed with 2XSSC, treated with RNase A (20µg/ml for 30 minutes at 37°C; USB Corporation, Cleveland, Ohio), 0.5X SSC for 30 minutes at 60°C, 0.1X SSC for 20 minutes and then rinsed in double distilled water. Slides were air dried and exposed to Biomax film (Kodak) for 3–6 weeks. To confirm the specificity of the signal observed with antisense riboprobes, controls used were sense riboprobes or RNase treatment (40µg/ml at 37°C for 30 minutes) prior to hybridization.

### Quantitation and data analysis

Densitometric analysis of LIM gene transcript levels was performed using the Macintosh-based Scion Imaging software (Scion, Frederick, Maryland, USA). Sections were observed directly on the monitor using a Sony 3 CCD color video camera (Model DXC-390P). ^14^C standards were used for calibration to correct for non-linearity. An equivalent area was outlined for each of the hippocampal subfields and optical density measurements from both hemispheres of 3–4 individual sections from each animal were analysed to calculate the mean value. Results were subjected to statistical Student’s t-test. Significance was determined at p < 0.05 using GraphPad inSTAT (version 3.05, LaJolla, California, USA). The following numbers of animals were used for each condition: Control adults, n = 7 ( *Clim1a*), 9 ( *Clim2*), 5 ( *Lhx1*), 5 ( *Lhx2*), 5 ( *Lhx9*), 4 ( *Lmo3*), 9 ( *Lmo4*). Kainate treated adults, n = 8 ( *Clim1a*), 8 ( *Clim2*), 5 ( *Lhx1*), 4 ( *Lhx2*), 4 ( *Lhx9*), 3 ( *Lmo3*), 8 ( *Lmo4*). Control pups, n = 6 ( *Clim1a*), 8 ( *Clim2*), 6 ( *Lhx1*), 8 ( *Lhx2*), 5 ( *Lhx9*), 7 ( *Lmo3*), 7 ( *Lmo4*). Kainate treated pups, n = 8 ( *Clim1a*), 8 ( *Clim2*), 8 ( *Lhx1*), 8 ( *Lhx2*), 7 ( *Lhx9*), 8 ( *Lmo3*), 7 ( *Lmo4*).

## References

[1] Guillemot F: Cell fate specification in the mammalian telencephalon. *Prog Neurobiol.* 2007;83(1):37–52 10.1016/j.pneurobio.2007.02.009 17517461

[2] Polleux F Ince-Dunn G Ghosh A: Transcriptional regulation of vertebrate axon guidance and synapse formation. *Nat Rev Neurosci.* 2007;8(5):331–40 10.1038/nrn2118 17453014

[3] Nóbrega-Pereira S Marín O: Transcriptional control of neuronal migration in the developing mouse brain. *Cereb Cortex.* 2009;19(Suppl 1):i107–13 10.1093/cercor/bhp044 19357392

[4] Lonze BE Ginty DD: Function and regulation of CREB family transcription factors in the nervous system. *Neuron.* 2002;35(4):605–23 10.1016/S0896-6273(02)00828-0 12194863

[5] Parrish JZ Emoto K Kim MD: Mechanisms that regulate establishment, maintenance, and remodeling of dendritic fields. *Annu Rev Neurosci.* 2007;30:399–423 10.1146/annurev.neuro.29.051605.112907 17378766

[6] West AE Greenberg ME: Neuronal activity-regulated gene transcription in synapse development and cognitive function. *Cold Spring Harb Perspect Biol.* 2011;3(6):a005744 10.1101/cshperspect.a005744 21555405 PMC3098681

[7] Lundgren SE Callahan CA Thor S: Control of neuronal pathway selection by the Drosophila LIM homeodomain gene apterous. *Development.* 1995;121(6):1769–73 7600992 10.1242/dev.121.6.1769

[8] Porter FD Drago J Xu Y: Lhx2, a LIM homeobox gene, is required for eye forebrain, and definitive erythrocyte development. *Development.* 1997;124(15):2935–4 9247336 10.1242/dev.124.15.2935

[9] Thor S Andersson SG Tomlinson A: A LIM-homeodomain combinatorial code for motor-neuron pathway selection. *Nature.* 1999;397(6714):76–80 10.1038/16275 9892357

[10] Ando H Kobayashi M Tsubokawa T: Lhx2 mediates the activity of Six3 in zebrafish forebrain growth. *Dev Biol.* 2005;287(2):456–468 10.1016/j.ydbio.2005.09.023 16226737

[11] Mangale VS Hirokawa KE Satyaki PR: Lhx2 selector activity specifies cortical identity and suppresses hippocampal organizer fate. *Science.* 2008;319(5861):304–309 10.1126/science.1151695 18202285 PMC2494603

[12] Milan M Cohen SM: Regulation of LIM homeodomain activity *in vivo*: a tetramer of dLDB and apterous confers activity and capacity for regulation by dLMO. *Mol Cell.* 1999;4(2):267–273 10.1016/S1097-2765(00)80374-3 10488342

[13] Thaler JP Lee SK Jurata LW: LIM factor Lhx3 contributes to the specification of motor neuron and interneuron identity through cell-type-specific protein-protein interactions. *Cell.* 2002;110(2):237–249 10.1016/S0092-8674(02)00823-1 12150931

[14] Milan M Diaz-Benjumea FJ Cohen SM: Beadex encodes an LMO protein that regulates Apterous LIM-homeodomain activity in Drosophila wing development: a model for LMO oncogene function. *Genes Dev.* 1998;12(18):2912–2920 10.1101/gad.12.18.2912 9744867 PMC317163

[15] Weihe U Milan M Cohen SM: Regulation of Apterous activity in Drosophila wing development. *Development.* 2001;128(22):4615–4622 11714686 10.1242/dev.128.22.4615

[16] Bulchand S Subramanian L Tole S: Dynamic spatiotemporal expression of LIM genes and cofactors in the embryonic and postnatal cerebral cortex. *Dev Dyn.* 2003;226(3):460–469 10.1002/dvdy.10235 12619132

[17] Bulchand S Grove EA Porter FD: LIM-homeodomain gene Lhx2 regulates the formation of the cortical hem. *Mech Dev.* 2001;100(2):165–175 10.1016/S0925-4773(00)00515-3 11165475

[18] Subramanian L Sarkar A Shetty AS: Transcription factor Lhx2 is necessary and sufficient to suppress astrogliogenesis and promote neurogenesis in the developing hippocampus. *Proc Natl Acad Sci U S A.* 2011;108(27):E265–74 10.1073/pnas.1101109108 21690374 PMC3131330

[19] Zhao Y Sheng HZ Amini R: Control of hippocampal morphogenesis and neuronal differentiation by the LIM homeobox gene Lhx5. *Science.* 1999;284(5417):1155–8 10.1126/science.284.5417.1155 10325223

[20] Yan CH Levesque M Claxton S: Lmx1a and lmx1b function cooperatively to regulate proliferation, specification, and differentiation of midbrain dopaminergic progenitors. *J Neurosci.* 2011;31(35):12413–25 10.1523/JNEUROSCI.1077-11.2011 21880902 PMC6703256

[21] Lakhina V Falnikar A Bhatnagar L: Early thalamocortical tract guidance and topographic sorting of thalamic projections requires LIM-homeodomain gene Lhx2. *Dev Biol.* 2007;306(2):703–13 10.1016/j.ydbio.2007.04.007 17493606

[22] Marcos-Mondéjar P Peregrín S Li JY: The lhx2 transcription factor controls thalamocortical axonal guidance by specific regulation of robo1 and robo2 receptors. *J Neurosci.* 2012;32(13):4372–85 10.1523/JNEUROSCI.5851-11.2012 22457488 PMC6622047

[23] Hobert O Tessmar K Ruvkun G: The Caenorhabditis elegans lim-6 LIM homeobox gene regulates neurite outgrowth and function of particular GABAergic neurons. *Development.* 1999;126(7):1547–1562 10068647 10.1242/dev.126.7.1547

[24] Manetopoulos C Hansson A Karlsson J: The LIM-only protein LMO4 modulates the transcriptional activity of HEN1. *Biochem Biophys Res Commun.* 2003;307(4):891–9 10.1016/S0006-291X(03)01298-1 12878195

[25] Elliott RC Khademi S Pleasure SJ: Differential regulation of basic helix-loop-helix mRNAs in the dentate gyrus following status epilepticus. *Neuroscience.* 2001;106(1):79–88 10.1016/S0306-4522(01)00198-1 11564418

[26] Elliott RC Miles MF Lowenstein DH: Overlapping microarray profiles of dentate gyrus gene expression during development- and epilepsy-associated neurogenesis and axon outgrowth. *J Neurosci.* 2003;23(6):2218–27 12657681 10.1523/JNEUROSCI.23-06-02218.2003 PMC6742005

[27] Takasu MA Dalva MB Zigmond RE: Modulation of NMDA receptor-dependent calcium influx and gene expression through EphB receptors. *Science.* 2002;295(5554):491–5 10.1126/science.1065983 11799243

[28] Parent JM Lowenstein DH: Seizure-induced neurogenesis: are more new neurons good for an adult brain? *Prog Brain Res.* 2002;135:121–131 10.1016/S0079-6123(02)35012-X 12143334

[29] Nadler JV: The recurrent mossy fiber pathway of the epileptic brain. *Neurochem Res.* 2003;28(11):1649–58 10.1023/A:1026004904199 14584819

[30] Overstreet-Wadiche LS Bromberg DA Bensen AL: Seizures accelerate functional integration of adult-generated granule cells. *J Neurosci.* 2006;26(15):4095–103 10.1523/JNEUROSCI.5508-05.2006 16611826 PMC6673901

[31] Scharfman HE McCloskey DP: Postnatal neurogenesis as a therapeutic target in temporal lobe epilepsy. *Epilepsy Res.* 2009;85(2–3):150–61 10.1016/j.eplepsyres.2009.03.006 19369038 PMC2713813

[32] Sperber EF Haas KZ Stanton PK: Resistance of the immature hippocampus to seizure-induced synaptic reorganization. *Brain Res Dev Brain Res.* 1991;60(1):88–93 10.1016/0165-3806(91)90158-F 1717181

[33] Lynch M Sayin U Bownds J: Long-term consequences of early postnatal seizures on hippocampal learning and plasticity. *Eur J Neurosci.* 2000;12(7):2252–64 10.1046/j.1460-9568.2000.00117.x 10947804

[34] Porter BE: Neurogenesis and epilepsy in the developing brain. *Epilepsia.* 2008;49(Suppl 5):50–4 10.1111/j.1528-1167.2008.01637.x 18522600 PMC2700768

[35] Alberini CM: Transcription factors in long-term memory and synaptic plasticity. *Physiol Rev.* 2009;89(1):121–45 10.1152/physrev.00017.2008 19126756 PMC3883056

[36] Hinks GL Shah B French SJ: Expression of LIM protein genes Lmo1, Lmo2, and Lmo3 in adult mouse hippocampus and other forebrain regions: differential regulation by seizure activity. *J Neurosci.* 1997;17(14):5549–59 9204936 10.1523/JNEUROSCI.17-14-05549.1997 PMC6793804

[37] Altman J Das GD: Autoradiographic and histological evidence of postnatal hippocampal neurogenesis in rats. *J Comp Neurol.* 1965;124(3):319–35 10.1002/cne.901240303 5861717

[38] Guéneau G Privat A Drouet J: Subgranular zone of the dentate gyrus of young rabbits as a secondary matrix. A high-resolution autoradiographic study. *Dev Neurosci.* 1982;5(4):345–58 10.1159/000112694 7140583

[39] Eckenhoff MF Rakic P: Nature and fate of proliferative cells in the hippocampal dentate gyrus during the life span of the rhesus monkey. *J Neurosci.* 1988;8(8):2729–47 3411351 10.1523/JNEUROSCI.08-08-02729.1988 PMC6569394

[40] Gray WP Sundstrom LE: Kainic acid increases the proliferation of granule cell progenitors in the dentate gyrus of the adult rat. *Brain Res.* 1998;790(1–2):52–9 10.1016/S0006-8993(98)00030-4 9593820

[41] Wenzel HJ Woolley CS Robbins CA: Kainic acid-induced mossy fiber sprouting and synapse formation in the dentate gyrus of rats. *Hippocampus.* 2000;10(3):244–60 10.1002/1098-1063(2000)10:3<244::AID-HIPO5>3.0.CO;2-7 10902894

[42] Lein ES Zhao X Gage FH: Defining a molecular atlas of the hippocampus using DNA microarrays and high-throughput *in situ* hybridization. *J Neurosci.* 2004;24(15):3879–89 10.1523/JNEUROSCI.4710-03.2004 15084669 PMC6729356

[43] Dong H Csernansky CA Goico B: Hippocampal neurogenesis follows kainic acid-induced apoptosis in neonatal rats. *J Neurosci.* 2003;23(5):1742–9 12629178 10.1523/JNEUROSCI.23-05-01742.2003 PMC6741957

[44] Okazaki MM Evenson DA Nadler JV: Hippocampal mossy fiber sprouting and synapse formation after status epilepticus in rats: visualization after retrograde transport of biocytin. *J Comp Neurol.* 1995;352(4):515–34 10.1002/cne.903520404 7721998

[45] Smith BN Dudek FE: Short- and long-term changes in CA1 network excitability after kainate treatment in rats. *J Neurophysiol.* 2001;85(1):1–9 11152700 10.1152/jn.2001.85.1.1

[46] Parent JM Yu TW Leibowitz RT: Dentate granule cell neurogenesis is increased by seizures and contributes to aberrant network reorganization in the adult rat hippocampus. *J Neurosci.* 1997;17(10):3727–38 9133393 10.1523/JNEUROSCI.17-10-03727.1997 PMC6573703

[47] Jiang M Lee CL Smith KL: Spine loss and other persistent alterations of hippocampal pyramidal cell dendrites in a model of early-onset epilepsy. *J Neurosci.* 1998;18(20):8356–68 9763479 10.1523/JNEUROSCI.18-20-08356.1998 PMC6792859

[48] Ribak CE Navetta MS: An immature mossy fiber innervation of hilar neurons may explain their resistance to kainate-induced cell death in 15–day-old rats. *Brain Res Dev Brain Res.* 1994;79(1):47–62 10.1016/0165-3806(94)90048-5 8070064

[49] Cornejo BJ Mesches MH Coultrap S: A single episode of neonatal seizures permanently alters glutamatergic synapses. *Ann Neurol.* 2007;61(5):411–26 10.1002/ana.21071 17323345

[50] Cross DJ Cavazos JE: Synaptic reorganization in subiculum and CA3 after early-life status epilepticus in the kainic acid rat model. *Epilepsy Res.* 2007;73(2):156–65 10.1016/j.eplepsyres.2006.09.004 17070016 PMC1876715

[51] Gray WP May K Sundstrom LE: Seizure induced dentate neurogenesis does not diminish with age in rats. *Neurosci Lett.* 2002;330(3):235–8 10.1016/S0304-3940(02)00810-8 12270636

[52] Bender RA Dubé C Gonzalez-Vega R: Mossy fiber plasticity and enhanced hippocampal excitability, without hippocampal cell loss or altered neurogenesis, in an animal model of prolonged febrile seizures. *Hippocampus.* 2003;13(3):399–412 10.1002/hipo.10089 12722980 PMC2927853

[53] Liu H Kaur J Dashtipour K: Suppression of hippocampal neurogenesis is associated with developmental stage, number of perinatal seizure episodes, and glucocorticosteroid level. *Exp Neurol.* 2003;184(1):196–213 10.1016/S0014-4886(03)00207-3 14637092

[54] Watanabe Y Johnson RS Butler LS: Null mutation of c-fos impairs structural and functional plasticities in the kindling model of epilepsy. *J Neurosci.* 1996;16(12):3827–36 8656277 10.1523/JNEUROSCI.16-12-03827.1996 PMC6578612

[55] Kashani AH Qiu Z Jurata L: Calcium activation of the LMO4 transcription complex and its role in the patterning of thalamocortical connections. *J Neurosci.* 2006;26(32):8398–408 10.1523/JNEUROSCI.0618-06.2006 16899735 PMC6673794

[56] Pennypacker KR McMillian MK Douglass J: Ontogeny of kainate-induced gene expression in rat hippocampus. *J Neurochem.* 1994;62(2):438–44 10.1046/j.1471-4159.1994.62020438.x 8294905

[57] Tauck DL Nadler JV: Evidence of functional mossy fiber sprouting in hippocampal formation of kainic acid-treated rats. *J Neurosci.* 1985;5(4):1016–22 3981241 10.1523/JNEUROSCI.05-04-01016.1985 PMC6565006

[58] Represa A Jorquera I Le Gal La Salle G: Epilepsy induced collateral sprouting of hippocampal mossy fibers: does it induce the development of ectopic synapses with granule cell dendrites? *Hippocampus.* 1993;3(3):257–68 10.1002/hipo.450030303 8353609

[59] Lynch M Sutula T: Recurrent excitatory connectivity in the dentate gyrus of kindled and kainic acid-treated rats. *J Neurophysiol.* 2000;83(2):693–704 10669485 10.1152/jn.2000.83.2.693

[60] Perez Y Morin F Beaulieu C: Axonal sprouting of CA1 pyramidal cells in hyperexcitable hippocampal slices of kainate-treated rats. *Eur J Neurosci.* 1996;8(4):736–748 10.1111/j.1460-9568.1996.tb01259.x 9081625

[61] Lee SK Pfaff SL: Synchronization of neurogenesis and motor neuron specification by direct coupling of bHLH and homeodomain transcription factors. *Neuron.* 2003;38(5):731–45 10.1016/S0896-6273(03)00296-4 12797958

[62] Xu B Li S Brown A: EphA/ephrin-A interactions regulate epileptogenesis and activity-dependent axonal sprouting in adult rats. *Mol Cell Neurosci.* 2003;24(4):984–99 10.1016/j.mcn.2003.08.003 14697663

[63] Chen HH Schock SC Xu J: Extracellular ATP-dependent upregulation of the transcription cofactor LMO4 promotes neuron survival from hypoxia. *Exp Cell Res.* 2007;313(14):3106–16 10.1016/j.yexcr.2007.04.026 17524392

[64] Nair A Vadodaria KC Banerjee SB: Stressor-specific regulation of distinct brain-derived neurotrophic factor transcripts and cyclic AMP response element-binding protein expression in the postnatal and adult rat hippocampus. *Neuropsychopharmacology.* 2007;32(7):1504–19 10.1038/sj.npp.1301276 17164818

