# Supplementary figures and images for: Seizure evoked regulation of LIM-HD genes and co-factors in the postnatal and adult hippocampus
Source: F1000Res. 2013 Oct 4;2:205. [Version 1] doi: 10.12688/f1000research.2-205.v1 (PMC4111125; doi:10.12688/f1000research.2-205.v1)

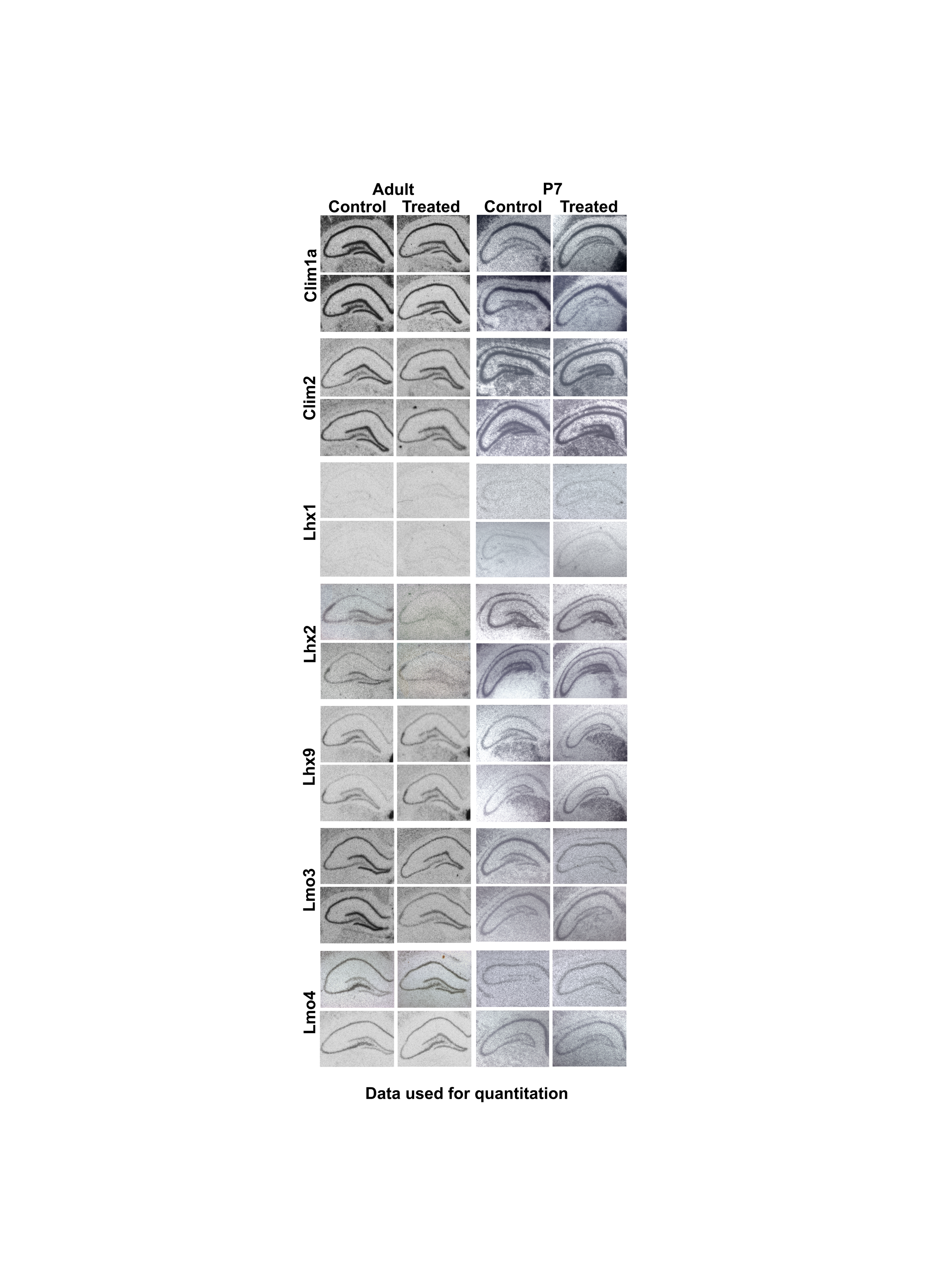

Supplement: Seizure evoked regulation of LIM genes in the hippocampus — Data figure. Expression of LIM genes and co-factors used for densitometric analysis Representative images of sections of brains from control and kainate-administered animals processed for radioactive in-situ hybridization of LIM-homeodomain genes in the hippocampus (in addition to Figure 2). Two sections from each condition are shown for adult and P7 animals. Dataset 1. Quantitations for Clim1a in the adult hippocampus Quantitations from densitometric analysis of hippocampal areas marked in Figure 2a from sections of brains from control and kainate-administered (treated) animals processed for radioactive in-situ hybridization of Clim1a in the adult hippocampus. Dataset 2. Quantitations for Clim2 in the adult hippocampus Quantitations from densitometric analysis of hippocampal areas marked in Figure 2a from sections of brains from control and kainate-administered (treated) animals processed for radioactive in-situ hybridization of Clim2 in the adult hippocampus. Dataset 3. Quantitations for Lhx1 in the adult hippocampus Quantitations from densitometric analysis of hippocampal areas marked in Figure 2a from sections of brains from control and kainate-administered (treated) animals processed for radioactive in-situ hybridization of Lhx1 in the adult hippocampus. Dataset 4. Quantitations for Lhx2 in the adult hippocampus Quantitations from densitometric analysis of hippocampal areas marked in Figure 2a from sections of brains from control and kainate-administered (treated) animals processed for radioactive in-situ hybridization of Lhx2 in the adult hippocampus. Dataset 5. Quantitations for Lhx9 in the adult hippocampus Quantitations from densitometric analysis of hippocampal areas marked in Figure 2a from sections of brains from control and kainate-administered (treated) animals processed for radioactive in-situ hybridization of Lhx9 in the adult hippocampus. Dataset 6. Quantitations for Lmo3 in the adult hippocampus Quantitations from densitometric analysis of hippocam [file f1000research-2-1764-s0000.tgz › Data.tif]
