# Supplementary material for: Seizure evoked regulation of LIM-HD genes and co-factors in the postnatal and adult hippocampus
Source: F1000Res. 2013 Oct 4;2:205. [Version 1] doi: 10.12688/f1000research.2-205.v1 (PMC4111125; doi:10.12688/f1000research.2-205.v1)
Supplement: Peer review history

# Peer review: Referee response for version 1

**DOI:** 10.5256/f1000research.1764.r4163

**Published:** 2014-03-28

**Authors:** Stan Leung

**Affiliations:** 1 Department of Physiology and Pharmacology, University of Western Ontario, London, ON, Canada

The authors showed field-specific changes in expression of LIM-HD genes *Lhx1*, *Lhx2*, and *Lhx9*, LIM-only gene *Lmo4*, and cofactor *Clim1a *in the adult hippocampus at 6 hours after seizure induction by kainic acid. No significant changes in expression were observed after seizure induction with the same dose of kainic acid in rat pups of postnatal day 7 (P7).

The subfield-specific changes in expression after kainic acid seizures were clearly presented for different members of the LIM-HD/ LIM-only family of transcription factors.  This work extends previous studies that showed seizure-induced changes in expression of LIM-only genes in adult rats. The lack of expression change in P7 rat pups may be related to the lack of seizure-induced structural plasticity in immature animals, i.e., lack of cell death, mossy fiber sprouting and neurogenesis. Members of the LIM-HD family of transcription factors are necessary for different aspects of the development of the hippocampus. Thus, the changes in these transcription factors after seizures in adult rats may be related to seizure-induced neural plasticity, such as axon sprouting, progenitor proliferation, and changes in neuronal cell survival.

The central question of whether LIM-HD/ LIM-only transcription factors are causally related to the neural plasticity induced by kainic acid seizures remains unanswered. Are transient changes in these transcription factors, at ~6 hours after seizures, sufficient or necessary for the structural changes induced by kainic acid seizures? The design of the present study does not allow the consequences of kainic acid seizures to be studied, and not all adult rats induced with kainic acid seizures showed plasticity to the same extent. In fact, whether seizures are essential for the expression changes is not totally clear. In other studies, a single dose of kainic acid (intraperitoneal 10 mg/kg) may not induce seizures in all adult rats, and the 10 mg/kg i.p. given to P7 rats was >5 times the dose needed for seizures as reported by Lynch et al. ( *Eur J Neurosci*, 2000).  The authors should provide some justification of the kainic acid dose used, in terms of seizure induction in their adult and P7 rats.

*“Regulation”* in the title apparently means “changes in expression” rather than better control or lack of control of the genes and cofactors.

The results are presented in an organized manner.

I would prefer to see the sample sizes (Ns) in the figure legend rather than in the *“Quantitation and data analysis”* section.

The issue of whether seizures are necessary and sufficient for the expression changes can be further discussed. Perhaps other molecules implicated for seizure-induced plasticity, e.g., neurotrophins, should be mentioned.

Overall, the experimental study appears to be well done.

I have read this submission. I believe that I have an appropriate level of expertise to confirm that it is of an acceptable scientific standard.

# Peer review: Referee response for version 1

**DOI:** 10.5256/f1000research.1764.r2010

**Published:** 2013-10-11

**Authors:** Bin Chen, Matthew Eckler

**Affiliations:** 1 Molecular, Cell, and Developmental Biology, University of California Santa Cruz, Santa Cruz, CA, USA; 2 University of California at Santa Cruz

Lakhina *et al*. examine the effect of chemically induced seizure on the expression of Lim family members and their cofactors, specially within the rodent hippocampus. Using *in situ* hybridization to measure mRNA levels they demonstrate that transcription of Lim, Lmo and Clim family members is altered in a field-specific manner specifically in adult but not early postnatal (P7) rats. These interesting observations extend previous work on seizure induced changes in Lmo1-3 expression (Hinks *et al.*) and raise several important questions for future study:

1. How rapid and persistent are the changes in gene expression following kainic acid administration?
2. Do alterations in gene expression drive physical remodeling within hippocampal neurons or vise verse?
3. Are there field specific differences in the timing of transcriptional changes?
4. What are the functional consequences of these transcriptional changes?
5. It is interesting that changes in transcription were observed in adult but not juvenile animals, whose circuits generally believed to be more plastic. Might these transcriptional changes be a means by which to counteract decreasing circuit plasticity in adults?

We have read this submission. We believe that we have an appropriate level of expertise to confirm that it is of an acceptable scientific standard.
